# Irf2bp2a regulates terminal granulopoiesis through proteasomal degradation of Gfi1aa in zebrafish

**DOI:** 10.1371/journal.pgen.1009693

**Published:** 2021-08-05

**Authors:** Shuo Gao, Zixuan Wang, Luxiang Wang, Haihong Wang, Hao Yuan, Xiaohui Liu, Saijuan Chen, Zhu Chen, Hugues de Thé, Wenqing Zhang, Yiyue Zhang, Jun Zhu, Jun Zhou

**Affiliations:** 1 Shanghai Institute of Hematology, CNRS-LIA Hematology and Cancer, Sino-French Research Center for Life Sciences and Genomics, State Key Laboratory of Medical Genomics, Rui Jin Hospital Affiliated to Shanghai Jiao Tong University School of Medicine, Shanghai, P.R. China; 2 Department of hematology, Shanghai General Hospital Affiliated to Shanghai Jiao Tong University School of Medicine, Shanghai, P.R. China; 3 Université de Paris 7/INSERM/CNRS UMR 944/7212, Equipe Labellisée No. 11 Ligue Nationale Contre le Cancer, Hôpital St. Louis, Paris, France; 4 Division of Cell, Developmental and Integrative Biology, School of Medicine, South China University of Technology, Guangzhou, P.R. China; University of Pennsylvania School of Medicine, UNITED STATES

## Abstract

The ubiquitin-proteasome system plays important roles in various biological processes as it degrades the majority of cellular proteins. Adequate proteasomal degradation of crucial transcription regulators ensures the proper development of neutrophils. The ubiquitin E3 ligase of Growth factor independent 1 (GFI1), a key transcription repressor governing terminal granulopoiesis, remains obscure. Here we report that the deficiency of the ring finger protein Interferon regulatory factor 2 binding protein 2a (Irf2bp2a) leads to an impairment of neutrophils differentiation in zebrafish. Mechanistically, Irf2bp2a functions as a ubiquitin E3 ligase targeting Gfi1aa for proteasomal degradation. Moreover, *irf2bp2a* gene is repressed by Gfi1aa, thus forming a negative feedback loop between Irf2bp2a and Gfi1aa during neutrophils maturation. Different levels of GFI1 may turn it into a tumor suppressor or an oncogene in malignant myelopoiesis. Therefore, discovery of certain drug targets GFI1 for proteasomal degradation by IRF2BP2 might be an effective anti-cancer strategy.

## Author summary

GFI1 is a key transcriptional repressor in hematopoiesis. The E3 ubiquitin ligase targeting GFI1 for proteasomal degradation remains obscure for many years. Here we show that the deficiency of the ring finger protein Irf2bp2a significantly impairs neutrophil maturation in zebrafish. Mechanistic studies reveal for the first time that instead of being a canonical transcription corepressor, Irf2bp2a functions as an E3 mediating the proteasomal degradation of Gfi1aa in neutrophil development.

## Introduction

The ubiquitin-proteasome system (UPS) plays an important role in degrading the majority of cellular proteins [1]. Ubiquitination includes three steps in cascade: activation, conjugation, and ligation, which are performed by ubiquitin-activating enzymes (E1s), ubiquitin-conjugating enzymes (E2s), and ubiquitin ligases (E3s), respectively. This sequential cascade eventually marks substrate proteins for degradation via the 26S proteasome and a crucial role of E3s is the specific recognition of substrates [2]. 500–1000 ubiquitin E3 ligases exist in humans which are classified into four families: RING finger, HECT, U box, and PHD finger [2].

Human Interferon regulatory factor 2 binding protein 2 (IRF2BP2) belongs to IRF2BP family which is composed of three members—IRF2BP1, IRF2BP2, and IRF2BPL [3]. The highly conserved IRF2BP family is structurally characterized by an N-terminal C4 type zinc finger motif which mediates homo- or hetero-dimerization/multimerization between different IRF2BP family members, and a C-terminal C3HC4 type ring finger motif that interacts with partner proteins [3]. Mostly, IRF2BP2 was described as a transcription corepressor for multiple partners including IRF2, NFAT1, and ETO2 [4–6]. Apart from transcription cofactor, the presence of the ring finger also enables the IRF2BP family members to function as a ubiquitin E3 ligase [7].

Hematopoiesis is the process by which pluripotent hematopoietic stem cells (HSCs) proliferate and differentiate into a repertoire of mature blood cells [8]. Various growth factors that stimulate cell proliferation and transcription factors activate and/or repress lineage-specific genes cooperate to regulate hematopoiesis [9]. Zinc finger protein growth factor independent 1 (GFI1) is a transcriptional repressor that plays diverse roles in normal and malignant hematopoiesis [10]. The human *GFI1* encodes a 55-kD nuclear protein containing an N-terminal SNAG motif necessary for transcriptional repression, and six consecutive C-terminal zinc finger motifs indispensable for DNA binding and interaction with partners [11,12]. An intermediate domain exists between the SNAG and zinc finger motifs, which can also bind proteins [10]. Mutations in *GFI1* gene can result in severe congenital neutropenia (SCN), and nonimmune chronic idiopathic neutropenia of adults in an autosomal dominant manner, which enhances the predisposition to leukemias [13–15].

It has been reported that the protein level of GFI1 is regulated by UPS in different cellular processes [16,17]. During myeloid differentiation, GFI1 protein is rapidly degraded by the 26S proteasome in mature granulocytes, whereas it is much more stable in monocytes [16]. In neuronal cells, ATAXIN-1 (ATX1) was shown to interact with GFI1 and enhance its proteasomal degradation [17]. Yet, ATX1 has never been described as an E3 ubiquitin ligase. In HEK293 cells, the knockdown of endogenous *TRIAD1* could increase GFI1 ubiquitination, implying that TRIAD1 might compete with an unidentified E3 ubiquitin ligase that promotes GFI1 for proteasomal degradation [18]. In addition, ubiquitin ligase FBXW7 has been shown to mediate the phosphorylation-dependent ubiquitination and degradation of GFI1 in gastric cancer cells [19].

In the current study, we demonstrate that the deficiency of *irf2bp2a* significantly impairs the maturation of neutrophils in zebrafish. Mechanistic studies reveal for the first time that instead of being a canonical transcription corepressor, Irf2bp2a functions as a ubiquitin E3 ligase in neutrophils development. A series of *in vivo* and *in vitro* evidence indicate that the transcription repressor Gfi1aa is the protein substrate of Irf2bp2a. Moreover, *irf2bp2a* gene can be repressed by Gfi1aa, thus forming a negative feedback loop that ensures an adequate Gfi1aa level to maintain proper neutrophils differentiation.

## Results

### Generation of an *irf2bp2a*-deficient zebrafish line

Two *irf2bp2* paralogs named *irf2bp2a* and *irf2bp2b* exist in zebrafish, which are both expressed in myeloid cells [20]. Recently, we have demonstrated that the loss of *irf2bp2b* resulted in a bias in neutrophils-macrophage progenitors (NMPs) cell fate choice favoring macrophages at the expense of neutrophils [20]. Mechanistically, the promoter of *pu*.*1*, one primary determinant of NMPs cell fate choice, was directly repressed by SUMOylated Irf2bp2b. Once *irf2bp2b* was mutated, the aberrant upregulation of *pu*.*1* ultimately led to the biased myelopoiesis, and the injection of wild type *irf2bp2b* mRNA could effectively rescue the defective myelopoiesis [20]. Intriguingly, although the functional domains of zebrafish Irf2bp2a and Irf2bp2b are nearly identical, *irf2bp2a* mRNA could not display the same rescue effect as *irf2bp2b* mRNA [20], implying their functions could be distinct.

**Fig 1:**
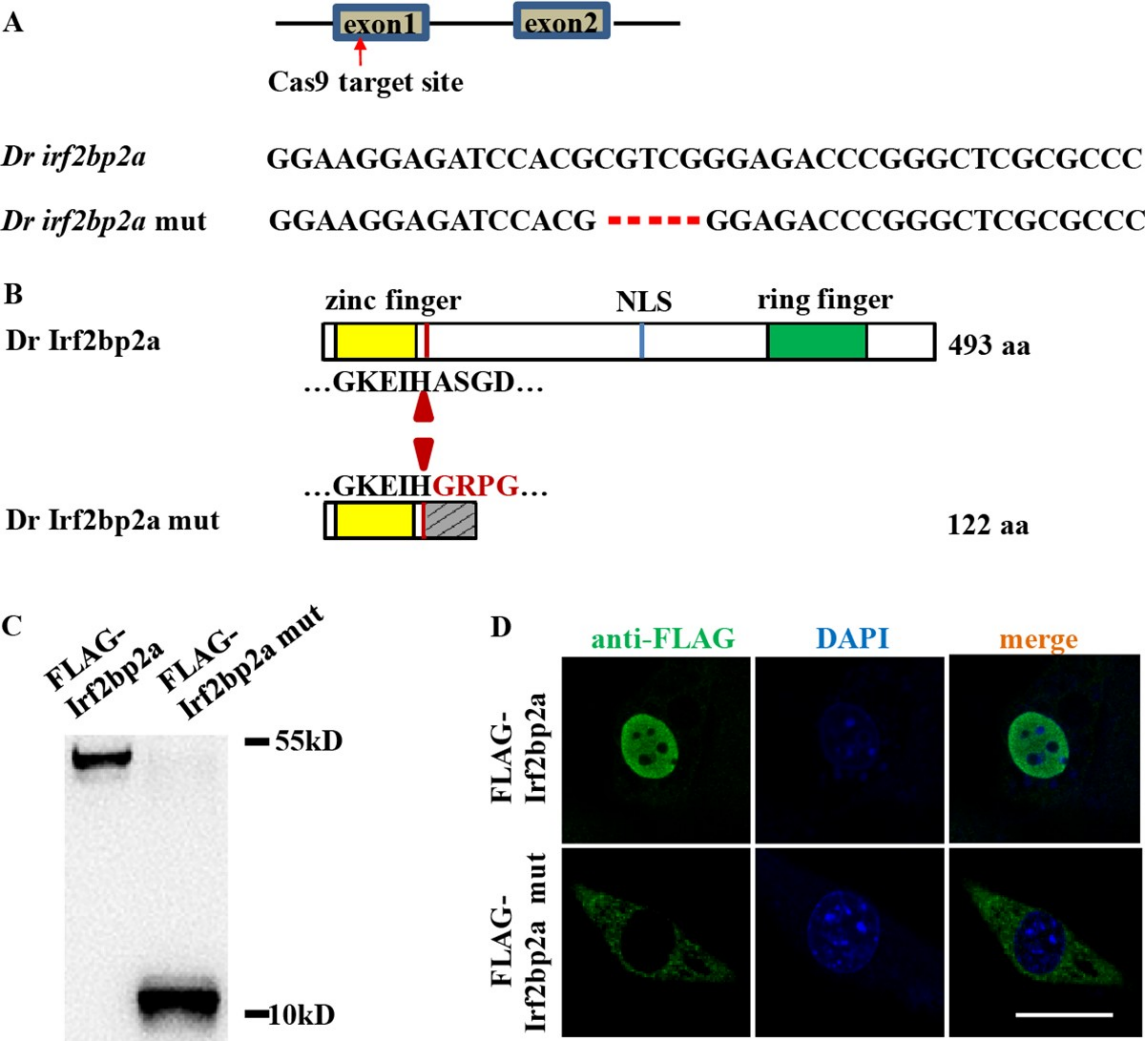
The establishment of a zebrafish *irf2bp2a* knockout line. (A) Schematic representation of Cas9 target site in the first exon of zebrafish *irf2bp2a*. The deleted nucleotides in the mutant gene are marked by hyphens. (B) Schematic representation of wild type (493 amino acids) and mutant Irf2bp2a proteins (122 amino acids). The site where the frameshift was introduced is marked by triangles. (C) Western blot analysis of FLAG-tagged wild type and mutant Irf2bp2a proteins expressed in HEK293 cells. (D) Immunofluorescence analysis of FLAG-tagged wild type (top panel) and mutant Irf2bp2a (bottom panel) proteins, demonstrating that the truncated protein lost its nuclear localization. Scale bar, 10 μm.

To elucidate the roles of *irf2bp2a* in hematopoiesis, an *irf2bp2a* mutant line was generated using the CRISPR/Cas9 system. Five nucleotides were deleted, which created a truncated protein containing only 122 amino acids by frameshifting (Fig 1A and 1B). A plasmid expressing the mutant *irf2bp2a* gene was transfected into HEK293 cells, and a short protein with predicted molecular weight was detected by western blot analysis (Fig 1C). Moreover, the Irf2bp2a mutant protein was shown to be located in the cytoplasm upon loss of the nuclear localization signal (NLS) [21] by immunofluorescence analysis (Fig 1D).

### Deficiency of zebrafish *irf2bp2a* specifically impairs neutrophils differentiation

Like mammalian hematopoiesis, zebrafish hematopoiesis is composed of primitive and definitive waves which emerge sequentially in distinct anatomical sites [22,23]. The rostral blood island (RBI) produces primitive myeloid cells [24], whereas the intermediate cell mass (ICM) gives rise to primitive erythrocytes and some neutrophils [25]. Definitive pluripotent HSCs generating all blood cell types arise in the ventral wall of the dorsal aorta (VDA), the zebrafish equivalent of the aorta/gonad/mesonephros (AGM) of mammals, then migrate through the caudal hematopoietic tissue (CHT) to the thymus and kidney marrow [26–29].

Whole-mount mRNA in situ hybridization (WISH) analysis conducted with a series of hematopoietic markers in *irf2bp2a*-deficient embryos revealed that the RBI derived primitive myeloid cells were unaffected (S1A–S1C’, S1N Fig). Meanwhile, the primitive erythrocytes originated from ICM also kept intact (S1D–S1H’ Fig). During definitive hematopoiesis, the normal specification of hematopoietic stem progenitor cells (HSPCs) was defined by the HSPCs related marker *c-myb* (S1M, S1M’, S1N Fig). Moreover, the preserved expression of erythroid, monocytic/macrophagic, and lymphoid markers, indicated these cells also developed normally (S1I–1L’, S1N Fig).

**Fig 2:**
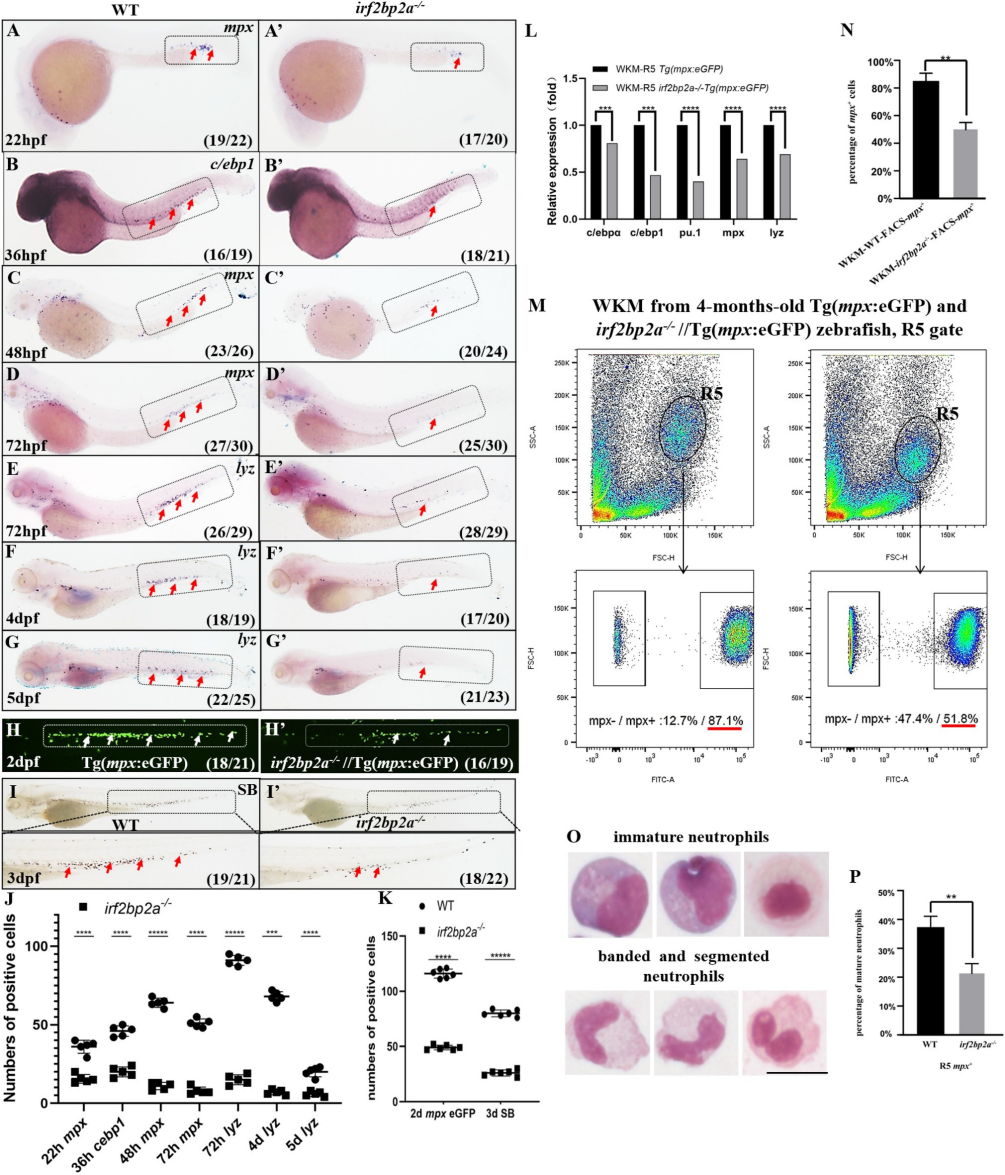
Deficiency of *irf2bp2a* specifically impairs neutrophils maturation in embryonic and adult zebrafish. (A-G’) WISH analyses of neutrophils markers *mpx* (A, A’, C-D’), *c/ebp1* (B, B’), *lyz* (E-G’) from 22 hpf to 5 dpf in wild type (WT) and *irf2bp2a*-deficient embryos. Grey boxes and red arrows indicate the main position of positive cells for each marker. n/n, number of embryos showing representative phenotype/total number of embryos examined. (H, H’) GFP positive cells are decreased in *irf2bp2a*^*-/-*^//*Tg(mpx*:*eGFP)* embryos at 2 dpf. (I, I’) Sudan Black positive cells are reduced in *irf2bp2a*-deficient embryos at 3 dpf. (J) Statistical results for A-G’ (Student t test, N = 5, 16–30 embryos were used for each probe. Each dot represents the mean value of one experiment, which was obtained from the counts of all of the embryos in the same group. Error bars represent mean ± SEM. ***P < 0.001, ****P <0.0001, *****P < 0.00001). (K) Statistical results for H-I’ (Student t test, N = 6, 16–22 embryos were used for each experiment. Each dot represents the mean value of one experiment. Error bars represent mean ± SEM. ****P < 0.0001, *****P <0.00001). (L) Quantitative reverse transcriptase polymerase chain reaction analysis of neutrophils differentiation-related genes in GFP positive cells enriched from *Tg(mpx*:*eGFP)* and *irf2bp2a*^*-/-*^//*Tg(mpx*:*eGFP)* embryos at 2 dpf. To determine the relative expression rate, data were normalized to the expression level of WT groups (which were set to 1.0) after normalized to the internal control of *β-actin* (Student t test, N = 5. Error bars represent mean ± SEM. ***P < 0.001, ****P < 0.0001). (M) FACS analysis of GFP positive cells within the R5 gate of WKMs in four-month-old wild type *Tg(mpx*:*eGFP)* (left panel) and *irf2bp2a*^*-/-*^//*Tg(mpx*:*eGFP)* zebrafish (right panel). (N) Statistical results for M in wild type *Tg(mpx*:*eGFP)* and *irf2bp2a*^*-/-*^//*Tg(mpx*:*eGFP)* zebrafish. (Student t test, N = 5, each time 1 male and 1 female were used in the WT group and 2 males and 2 females were used in the mutant group. Error bars represent mean ± SEM. **P < 0.01). (O) May-Grünwald Giemsa staining of *mpx*^*+*^ neutrophilss isolated from R5 gate of *irf2bp2a*^*-/-*^//*Tg(mpx*:*eGFP)* mutants and siblings. Scale bar, 10 μm. (P) 500 cytospin-collected GFP^*+*^ cells were counted on slides. Immature and mature neutrophils were distinguished and quantitated by morphology, and the proportion of mature neutrophils was compared between WT and mutant groups (Student t test, N = 3. Error bars represent mean ± SEM. **P < 0.01).

The only defect observed in *irf2bp2a*-deficient embryos was restricted to neutrophils lineage. A dramatic reduction of several neutrophil markers including *myeloperoxidase* (*mpx*) [30], *lysozyme C* (*lyz*) [24,31], and *c/ebp1*[24,32], was observed from 22 hours post-fertilization (hpf) to 5 days post-fertilization (dpf) by WISH (Fig 2A–2G’ and 2J). Such defective neutrophil development was further confirmed in *irf2bp2a*^*-/-*^//*Tg(mpx*:*eGFP)* embryos and by Sudan Black (SB) staining [24] (Fig 2H–2I’ and 2K). It is worth noting that the signal intensity per cell for GFP and SB staining of the neutrophils in *irf2bp2a*-deficient embryos was weaker than that in siblings, implying these neutrophils might not be fully differentiated. The remaining *mpx*^+^ cells were isolated from *irf2bp2a* mutant embryos by flow cytometry (FACS), and quantitative reverse transcription PCR (RT-qPCR) analysis revealed that the expression level of *c/ebpα*, *c/ebp1* and *pu*.*1* (critical transcription factors implicated in terminal granulopoiesis), as well as *mpx* and *lyz* (neutrophils granule proteins) were significantly reduced compared to wild type siblings (Fig 2L). Since *irf2bp2a*^*-/-*^ zebrafish could survive into adulthood, FACS analyses were performed with the whole kidney marrow (WKM) samples harvested from wild type *Tg(mpx*:*eGFP)* and *irf2bp2a*^-/-^//*Tg(mpx*:*eGFP)* lines in adults. Myeloid population (R5 gate) could be distinguished based on its characteristic scatter profile. Not only the percentage of *mpx*^+^ cells in R5 gate (87.1% vs 51.8%), but also the signal intensity of GFP were sharply decreased in *irf2bp2a*^-/-^//*Tg(mpx*:*eGFP)* zebrafish compared to wild type siblings by fluorescence analysis (Fig 2M and 2N). In line with these observations, similar decreased expression of *c/ebpα*, *c/ebp1*, *pu*.*1*, *mpx* and *lyz* was found in *mpx*^+^ cells isolated from R5 gate by RT-qPCR analysis (S2 Fig). Meanwhile, morphological analysis by May-Grünwald Giemsa staining displayed a profound reduction of mature neutrophils in *irf2bp2a* mutants (37.3% vs 21.6%) (Fig 2O and 2P). Combining the data above, a conclusion could be drawn that the differentiation of neutrophils was severely impaired in *irf2bp2a*^-/-^ zebrafish.

The injection with specific *irf2bp2a* morpholino (MO) could exactly phenocopy the aberrant neutrophils development presented in *irf2bp2a*^-/-^ embryos (S3 Fig). In addition, all of the impairments in *irf2bp2a*^-/-^ and morphant embryos could be effectively rescued with the wild type *irf2bp2a* mRNA, thus confirming the specificity of the defective phenotype.

### Irf2bp2a acts as a potential ubiquitin E3 ligase, rather than a canonical transcription corepressor, in regulating the development of neutrophils

IRF2BP2 was elucidated as a transcription corepressor in different biological systems. The C-terminal ring finger motif of IRF2BP2 mediates the binding with its interacting transcription factors [4,5], whereas the N-terminal zinc finger motif enables the homo or hetero-dimerization/multimerization between different IRF2BP2 family members [3]. In addition, since C4 zinc finger is found in DNA binding domain (DBD) of transcription factors including GATA, RAR and RXR [33,34], the possibility that IRF2BP2 functions as a transcription factor could not be ruled out. Recently, we have demonstrated that the C4 zinc finger is required for Irf2bp2b to repress *pu*.*1* expression through direct binding to its promoter [20]. Moreover, the post-translational modification—SUMOylation on a conserved lysine at the C-terminus of Irf2bp2b is pivotal for its transcriptional repression [20].

**Fig 3:**
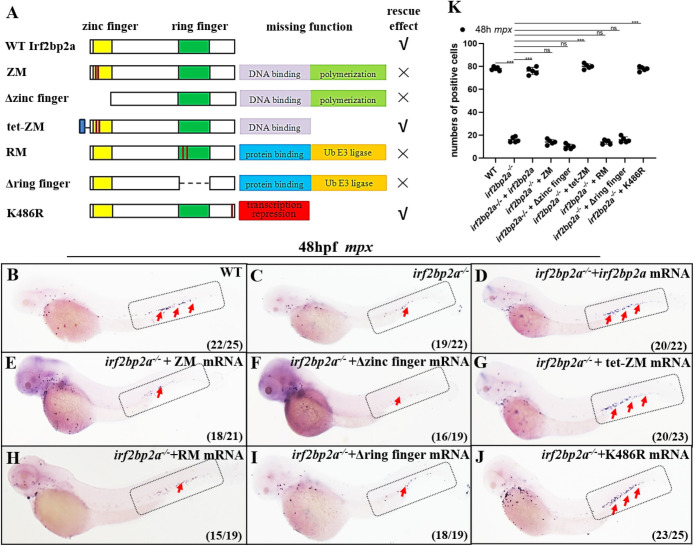
Polymerization and potential ubiquitin E3 ligase function are indispensable for Irf2bp2a in regulating the differentiation of neutrophilss. (A) Structure, missing function and rescue effect of wild type and variant forms of Irf2bp2a. (B-J) *Irf2bp2a* mRNA rescue assays in *irf2bp2a*^-/-^ embryos. *Mpx* probe was used in WISH to examine rescue effect with wild *irf2bp2a* (D), ZM (E), Δzinc finger (F), tet-ZM (G), RM (H), Δring finger (I) and K486R mutant (J) mRNA injections. (K) The statistical significance was calculated by using one-way ANOVA. The statistical significance was calculated using 1-way ANOVA followed by Dunnett T3 correction. The asterisk indicates a statistical difference (N = 5, 15–25 embryos were used for each experiment. Each dot represents the mean value of one experiment. Error bars represent mean ± SEM. ns: no statistical significance; ***P < 0.001).

To investigate how Irf2bp2a regulates neutrophils maturation, several critical cysteines within the C4 zinc finger and C3HC4 ring finger were mutated respectively (C14/17A and C409/413A, named ZM and RM thereafter) as previously reported [20] (Fig 3A). For the Irf2bp2a ZM mutant, the multimerization and DNA binding properties were both abolished. Based on it that, the tetramerization motif of human P53 (amino acids 324–355) was fused in frame (tet-ZM), therefore restoring the multimerization capacity of this mutant [20]. For the RM mutant, the interaction with partners was interrupted. We also constructed an Irf2bp2a K486R mutant, of which the conserved SUMOylation site was mutated, thus the repression capacity was abrogated.

*In vivo* rescue assays were performed with a series of mutant mRNAs as described above. The ZM mutant did not display any rescue effect, whereas the tet-ZM mutant had a significant rescue effect as wild type *irf2bp2a* mRNA (Fig 3B–3G and 3K). These results suggest that the polymerization, rather than the DNA binding property of the C4 zinc finger, is indispensable. Similar to the ZM mutant, RM mutant failed to rescue the defects in *irf2bp2a*^-/-^ zebrafish (Fig 3H and 3I and 3K). Nevertheless, the K486R mutant could efficiently rescue the defects (Fig 3J and 3K), implying that Irf2bp2a might no longer be a canonical transcription corepressor. Except protein binding, ring finger is a characteristic domain with ubiquitin E3 ligase activity, we thus postulated that Irf2bp2a would be a potential E3 on the regulation of n.

### Irf2bp2a mediates the ubiquitination and proteasomal degradation of transcription repressor Gfi1aa

C/EBPα, C/EBPε, and PU.1 are three critical transcription factors implicated in neutrophils maturation [35–37]. Since the transcription levels of *c/ebpα*, *c/ebp1* (the ortholog of *C/EBPε*) and *pu*.*1* were all downregulated in *irf2bp2a*-deficient neutrophils, certain suppressors might accumulate upon loss of Irf2bp2a, which in turn impedes the maturation of neutrophils.

**Fig 4:**
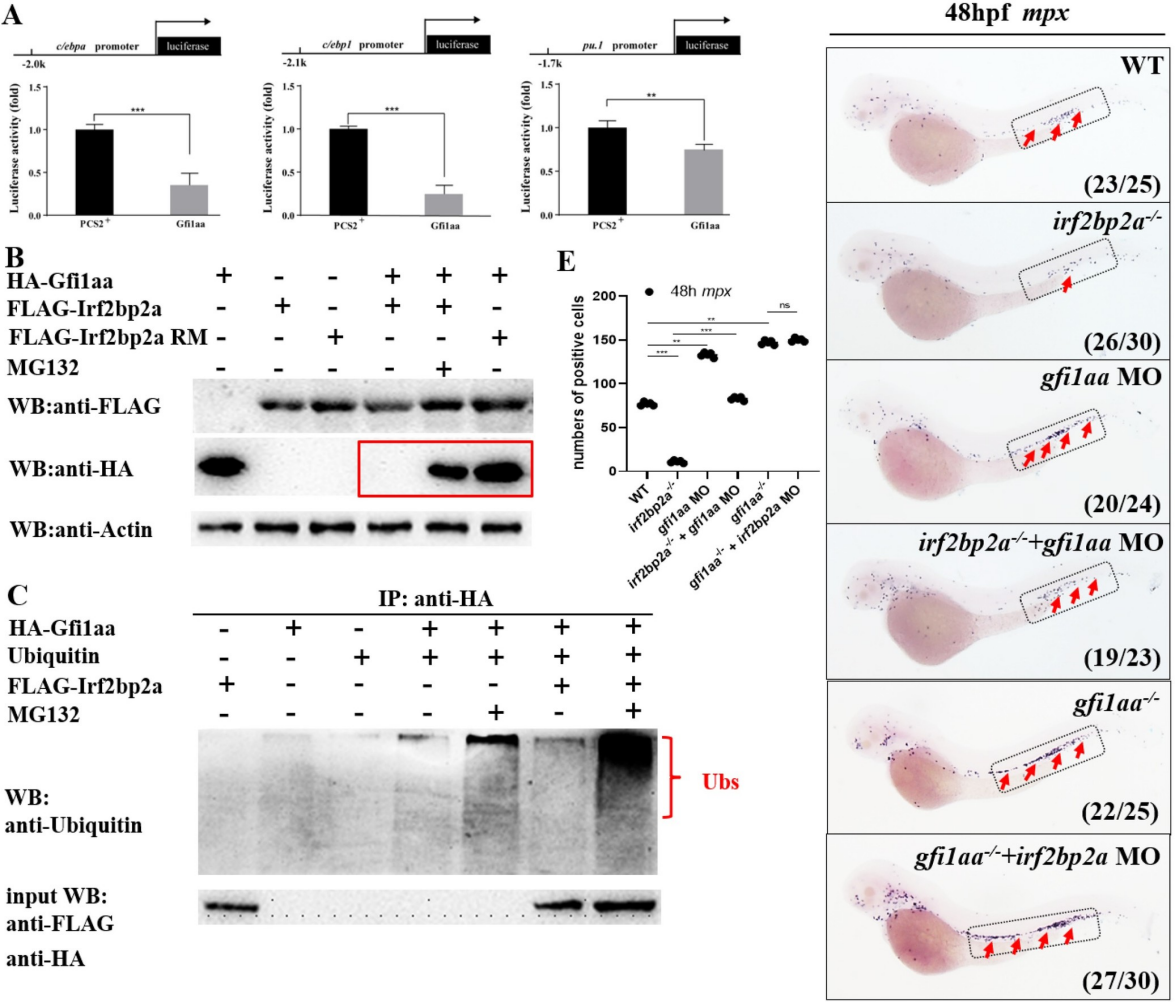
Irf2bp2a targets the transcription repressor Gfi1aa for ubiquitination and proteasomal degradation. (A) Luciferase reporter assay. Bars showed the relative luciferase activity on the zebrafish *c/ebpα* promoter (-2.0 kb), *c/ebp1* promoter (-2.1 kb), and *pu*.*1* promoter (-1.7 kb) (Student t test, N = 3. Error bars represent mean ± SEM. **P < 0.01, ***P < 0.001). (B) Western blot analysis (anti-FLAG and anti-HA) of FLAG-Irf2bp2a, FLAG-Irf2bp2a RM, and HA-Gfi1aa expressed in HEK293 cells. The proteasome inhibitor MG132 (2.5 μM) was used to inhibit the degradation of ubiquitinated proteins. Equal protein amounts for each sample were loaded (anti-Actin). (C) HA-Gfi1aa protein was immunoprecipitated (IP) with an anti-HA antibody from HEK293 cells co-expressing Ubiquitin and FLAG-Irf2bp2a. Many more adducts of Ubiquitin were detected by western blot with an anti-Ubiquitin antibody. (D) WISH assay of *mpx* in WT, *irf2bp2a*^*-/-*^ mutant embryos, wild type and *irf2bp2a*^*-/-*^ mutant embryos injected with *gfi1aa* MO, *gfi1aa*^*-/-*^ embryos, and *gfi1aa*^*-/-*^ embryos injected with *irf2bp2a* MO. (E) Statistic result for D. The statistical significance was calculated by using one-way ANOVA. The asterisk indicates a statistical difference (N = 5, 19–30 embryos were used for each experiment. Each dot represents the mean value of one experiment. Error bars represent mean ± SEM. **P < 0.01; ***P < 0.001).

Transcription repressor GFI1 is a master regulator involved in both normal myeloid development and MDS/AML pathogenesis [38]. *C/EBPα*, *C/EBPε* and *PU*.*1* have been demonstrated to be repressed by GFI1 in different cellular context [16,39–41]. We performed the luciferase activity assay on the promoters of zebrafish *c/ebpα*, *c/ebp1*, and *pu*.*1* [20] respectively. The results indicated that Gfi1aa exhibited a significant repression effect on all of the three promoters (Fig 4A).

Based on these observations, we postulated that Gfi1 is a bona fide substrate of Irf2bp2a. To test this hypothesis, the *gfi1aa* gene (the zebrafish ortholog of mammalian *GFI1*) [42] was first cloned into an HA-tagged expressing vector, and transfected into HEK293 cells with or without FLAG-tagged *irf2bp2a* plasmid co-transfection. Western blot analysis indicated that Gfi1aa protein disappeared in the presence of Irf2bp2a, but was restored when treated with proteasome inhibitor MG132 (Fig 4B). By contrast, Gfi1aa protein existed with co-transfection of Irf2bp2a RM without MG132 treatment (Fig 4B). Next, additional experiments were performed to further confirm the effect of Irf2bp2a on Gfi1aa ubiquitination and proteasomal degradation. HEK293 cells were transfected with HA-Gfi1aa, Ubiquitin (Ub), and with or without FLAG-Irf2bp2a. After immunoprecipitation (IP) with an anti-HA antibody, western blot detected by anti-Ubiquitin revealed that pull-downed Gfi1aa protein was intensively ubiquitinated in the presence of Irf2bp2a (Fig 4C), indicating Gfi1aa is a real protein substrate of Irf2bp2a for ubiquitination.

To further demonstrate that Irf2bp2a regulates neutrophils development through degrading Gfi1aa, a series of *in vivo* assays was carried out. An obvious rescue effect could be obtained with *gfi1aa* MO in *irf2bp2a* mutants (Fig 4D and 4E). Moreover, we took advantage of a zebrafish *gfi1aa* knockout line. Intriguingly, all of the neutrophil markers downregulated in our *irf2bp2a*^-/-^ embryos were found to be profoundly elevated in *gfi1aa*^-/-^ mutants (Figs 4D and 4E and S4). Note that no alleviation could be observed in *gfi1aa*^-/-^ embryos injected with *irf2bp2a* MO compared to *gfi1aa*^-/-^ mutant, implying that *gfi1aa* is the downstream of *irf2bp2a* (Fig 4D and 4E).

Taken together, these findings indicate that zebrafish Irf2bp2a functions as a ubiquitin E3 ligase of transcription repressor Gfi1aa in regulating the differentiation of neutrophils.

### *Irf2bp2a* gene is repressed by Gfi1aa

A series of neutrophil markers were decreased upon loss of *irf2bp2a*. By contrast, a reverse phenotype emerged once *irf2bp2a* mRNA was injected into wild type embryos. These observations suggested that the endogenous level of *irf2bp2a* must be tuned to a proper range to ensure normal neutrophil development. Since GFI1 is a potent transcription repressor, we questioned whether Gfi1aa could inhibit the expression of *irf2bp2a*.

**Fig 5:**
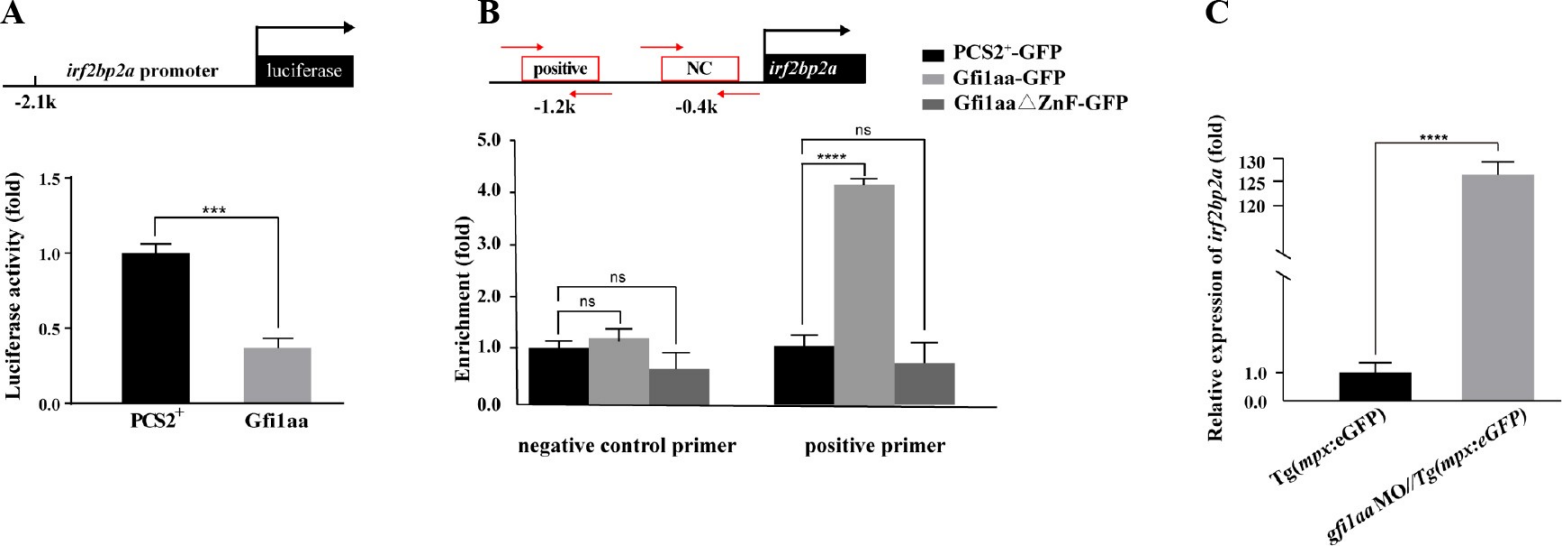
*Irf2bp2a* gene is repressed by Gfi1aa. (A) Luciferase reporter assay. Bars showed the relative luciferase activity on the zebrafish *irf2bp2a* promoter (-2.1 kb) (Student t test, N = 3. Error bars represent mean ± SEM. ***P < 0.001). (B) CHIP-PCR analysis of *irf2bp2a* promoter in zebrafish embryos expressing GFP, Gfi1aa-GFP or Gfi1aa-Δzinc finger-GFP using an anti-GFP antibody. The statistical significance was calculated by using one-way ANOVA. The asterisk indicates a statistical difference (N = 4. Error bars represent mean ± SEM. ns: not statistically significant, ****P < 0.0001). (C) *irf2bp2a* relative expression level analyzed by quantitative PCR in GFP-positive cells sorted from control *Tg(mpx*:*eGFP)* embryos and *gfi1aa* MO injected *Tg(mpx*:*eGFP)* morphants at 2 dpf (Student t test, N = 5. Error bars represent mean ± SEM. ****P < 0.0001).

To test this hypothesis, *irf2bp2a* promoter was analyzed by bioinformatics and a dozen of putative Gfi1 binding sites were predicted. The predicted zebrafish *irf2bp2a* -2.1 kb promoter was cloned into a luciferase reporter vector, and cotransfected with either an empty vector or a *gfi1aa* expressing vector. The result showed that the luciferase expression was significantly repressed by Gfi1aa (Fig 5A). Moreover, an *in vivo* chromatin immunoprecipitation PCR (CHIP-PCR) analysis was conducted in zebrafish embryos expressing GFP, Gfi1aa-GFP, or Gfi1aa Δzinc finger-GFP using an anti-GFP antibody. The results showed that the promoter region of *irf2bp2a* could be specifically co-immunoprecipitated (co-IP) with Gfi1aa-GFP (Fig 5B). In addition, *mpx*^*+*^ cells were enriched from *gfi1aa*-deficient embryos by FACS, and RT-qPCR analysis showed that the transcript level of *irf2bp2a* was much higher than that of siblings (Fig 5C).

In summary, the data suggests that a negative circuit exists in Irf2bp2a-Gfi1aa axis during zebrafish neutrophil differentiation.

## Discussion

Accumulating evidence indicates that proper turnover of cellular proteins plays important roles in normal and malignant hematopoiesis [43,44]. GFI1 is a key regulator implicated in multilineage hematopoietic cell development [10]. It has been reported that in granulocytes, the mRNA level of *GFI1* is high, whereas the protein level is relatively low due to rapid proteasomal degradation [16]. However, the specific ubiquitin E3 ligase mediating GFI1 degradation involved in granulopoiesis has not been identified. In the current study, we demonstrate that Irf2bp2a serves as an E3 ligase of Gfi1aa during zebrafish neutrophil differentiation. Although *irf2bp2a* is ubiquitously expressed in a variety of blood cell types, its expression in neutrophils is more prominent. In line with the expression pattern, only the development of neutrophils, but not other lineages, is affected in *irf2bp2a*-deficient zebrafish, which is reflected by a series of normal lineage specific markers in embryos and normal populations of erythrocytes (R1 and R2 gates), lymphocytes (R3 gate), and immature precursors (R4 gate) in adults (S5 Fig).

Ring finger of Irf2bp2a is a characteristic domain with ubiquitin E3 ligase activity. Since the ring finger domain in zebrafish Irf2bp2a and Irf2bp2b proteins is nearly identical, similar degradation effect on Gfi1aa was observed in HEK293 cells expressing Irf2bp2a or Irf2bp2b. However, the consequence of the *in vivo* deficiency of *irf2bp2a* extremely differs from that of *irf2bp2b* in myelopoiesis. While only neutrophil lineage is impaired in *irf2bp2a* mutants, neutrophils and macrophage lineages are simultaneously affected upon loss of *irf2bp2b*. Mechanistically, Irf2bp2a acts as an E3 of Gfi1aa in regulating the differentiation of neutrophils, whereas SUMOylated Irf2bp2b functions as a transcription repressor to inhibit *pu*.*1* expression in NMP cell fate choice [20]. Such discrepancy of Irf2bp2a and Irf2bp2b is evidenced by the different requirement of the ring finger domain and SUMOylation in *in vivo* rescue assays [20].

The neutrophil population is impaired in our *irf2bp2a*-deficient zebrafish, intriguingly, similar defect was also found in *Gfi1*-deficient mice [45]. Nevertheless, the mechanisms might be quite different. The observations from mice and humans [45,46] implied that the defective neutrophil population is probably due to a bias in NMPs cell fate in favor of macrophages at the expense of neutrophils, in which the expression of Gfi1 target genes such as *Egr1*/*2*, *Nab2*, *Csf1* and its receptor *Csf1r* are aberrantly increased. Yet, the defective neutrophil population in zebrafish is due to the accumulated non-degradable Gfi1aa protein, which in turn inhibits the expression of transcription factors involved in neutrophil maturation such as *c/ebpα*, *c/ebp1*, and *pu*.*1*.

In the human genome there is only one *IRF2BP2* gene, which produces two isoforms also known as *IRF2BP2a* and *IRF2BP2b* due to alternative splicing. Compared with IRF2BP2b, an additional sequence (sixteen amino acids long) is located within the intermediate domain of IRF2BP2a [47]. Like zebrafish Gfi1aa, human GFI1 was also degraded in HEK293 cells expressing either *IRF2BP2a* or *IRF2BP2b* (S6 Fig), suggesting a similar IRF2BP2/GFI1 relationship might exist in the human scenario. HL60, a human promyelocytic leukemia cell line, can be induced to differentiate toward granulocytes with ATRA. It has been reported that in an *in vitro* degradation experiment, ^35^S-labeled GFI1 protein could be efficiently degraded with lysate from HL60 cells treated with ATRA [16]. Our RT-qPCR analyses reveal that *IRF2BP2a* and *IRF2BP2b* are both expressed in HL60 cells, and their transcription levels are increasingly upregulated as cells differentiate (S7 Fig), which implies that similar GFI1 degradation events might also happen during terminal granulopoiesis in human cells.

Hundreds of E3 enzymes specifically recognize substrates that are destined for ubiquitination in a repertoire of cellular proteins [2]. Sometimes different ligases share the same substrate [48,49]. It has been reported that the knockdown of endogenous *TRIAD1* in HEK293 cells leads to increased GFI1 ubiquitination, implying that TRIAD1 can inhibit the turnover of GFI1 through competition with an unidentified E3 [18]. To elucidate whether IRF2BP2 is such unknown E3, we over-expressed *TRIAD1*, *GFI1*, and *IRF2BP2* in HEK293 cells. Even in the presence of abundant TRIAD1, GFI1 protein was still degraded by IRF2BP2 (S8 Fig). It has been also reported that the phosphorylated GFI1 is a substrate of SCF-type ubiquitin ligase FBXW7 in gastric cancer cells. All of these observations suggest that GFI1 would be targeted by different E3s, probably in a tissue specific manner.

## Materials and methods

### Ethics statement

The study was approved by the Ethics Committee of Rui Jin Hospital Affiliated to Shanghai Jiao Tong University School of Medicine. All animal work was approved by the Animal Care and Use Committee of Shanghai Jiao Tong University.

### Zebrafish maintenance and mutant generation

Zebrafish were raised, bred, and staged according to standard protocols [50]. The following strains were used: AB, *Tg(mpx*:*GFP)rj30* (ZFIN database). For CRISPR9 mediated *irf2bp2a* knockout zebrafish generation, guide RNA (gRNA) targeting exon1 of *irf2bp2a* was designed using an online tool ZiFiT Targeter software (http://zifit.partners.org/ZiFiT), which was synthesized by cloning the annealed oligonucleotides into the gRNA transcription vector. Cas9 mRNA and gRNA were co-injected into one-cell stage zebrafish embryos. The injected F0 founder embryos were raised to adulthood and then outcrossed with wild type zebrafish. F1 embryos carrying potential indel mutations were raised to adulthood. Then PCR amplification and sequencing were performed on genomic DNA isolated from tail clips of F1 zebrafish to identify mutants.

### Whole-mount in situ hybridization (WISH)

Digoxigenin-labeled RNA probes were transcribed with T7, T3 or SP6 polymerase (Ambion, Life Technologies, USA). WISH was performed as described previously [51]. The probes labeled by digoxigenin were detected using alkaline phosphatase coupled anti-digoxigenin Fab fragment antibody (Roche) with 5-bromo-4-chloro-3-indolyl-phosphate nitro blue tetrazolium staining (Vector Laboratories, Burlingame, CA, USA).

15–30 embryos were used for each probe. The positive signals were counted under a microscope, and the mean value was obtained from the counts of all of the embryos in the same group.

### Sudan Black staining

The embryos treated with 4% paraformaldehyde (PFA) overnight at 4°C were incubated with a Sudan Black (Sigma-Aldrich) solution for about 30 minutes to detect the granules of granulocytes. The detailed method was described previously [52]. Staining was then observed under a microscope.

### FACS analysis, cell collection and May-Grünwald-Giemsa staining

FACS analysis and cell collection were performed as described [53]. Wild type *Tg(mpx*:*eGFP)* and *irf2bp2a*^*-/-*^//*Tg(mpx*:*eGFP)* embryos were dissociated into single cells using 0.05% trypsin (Sigma) as previously described [54] at 48 hpf. These dissociated cells were passed through a 40-μm mesh, centrifuged at 450g, and suspended in 5% FBS/PBS before addition of propidium iodide to a final concentration of 1 μg/ml for exclusion of dead cells. Wild type zebrafish (without GFP) were used as blank to determine the background values in GPF-controls. The GFP^+^ cells of each group were collected from a total of ~3000 embryos using a FACS Vantage flow cytometer (Beckton Dickenson) (~1000 embryos once, performed 3 times). For the whole kidney marrow (WKM) samples, FACS analysis was based on forward and side scatter characteristics, propidium iodide exclusion and GFP fluorescence. The GFP^+^ cells in R5 gate was enriched from WKM samples of wild type *Tg(mpx*:*eGFP)* and *irf2bp2a*^*-/-*^//*Tg(mpx*:*eGFP)* zebrafish (from four to seven-month-old, two to four WKMs were collected from each zebrafish line of the same age, performed 3 times). Immature and mature neutrophils were counted by MGG staining with cytospin-collected cells on slides.

### Quantitative RT-PCR

The quantitative PCR was carried out with SYBR Green Real-time PCR Master Mix (TOYOBO) with ABI 7900HT real-time PCR machine and analyzed with Prism software. *β-actin* was served as the internal control. The primers used are listed in S1 Table. Each time a different batch of samples was used. The expression levels of each interested gene were normalized to internal control *β*-*actin* by real-time qPCR and compared with WT group which was set to 1.0. Real time qPCR was performed with gene specific primers and gene expression levels were analyzed by comparative CT method.

### Plasmid construction

Zebrafish *irf2bp2a* gene and its serial mutants were cloned into PCS2^+^ vector. For the luciferase reporter, the -2.0 kb promoter of zebrafish *c/ebpα* gene, -2.1 kb promoter of zebrafish *c/ebp1* gene, and the -2.1 kb promoter of zebrafish *irf2bp2a* gene were cloned into the PGL3 basic vector (Promega, Madison, WI, USA). Primers used were listed in S1 Table.

### Morpholino and mRNA synthesis for microinjection

Zebrafish *irf2bp2a* (5’-ACGACATCGCTCTCTCTCGGGCGAA-3’) and *gfi1aa* (5’-GTAAACATGCCGAGGTCATTTTTGG-3’) morpholino oligonucleotides (MO) targeting the transcriptional initiation ATG of *irf2bp2a* and *gfi1aa* was designed and purchased from Gene Tools. Full-length capped mRNA samples were all synthesized from linearized plasmids using the mMessage mMachine SP6 kit (Invitrogen, Thermo Fisher, USA). Microinjection concentration of mRNA was between 50–200 ng/μl and 2 nl of mRNA was injected at one-cell stage embryos. All injections were performed with a Harvard Apparatus micro-injector.

### Cell culture and luciferase reporter assay

HEK293 cells were maintained in DMEM (Life technologies, Grand Island, NY, USA) with 10% Fetal Bovine Serum (Life technologies, Grand Island, NY, USA). Plasmid transfection was carried out with Effectene Transfection Reagent (QIAGEN) according to manufacturer’s instruction. For the luciferase reporter assay, cells were harvested 48 hours after transfection and analyzed using the Dual Luciferase Reporter Assay Kit (Promega, Maddison, WI, USA), according to the manufacturer’s protocols. Primers used were listed in S1 Table.

### Western blot and co-immunoprecipitation assay

HEK293 cells, which had been transfected with plasmids for 48 hours, were washed with phosphate-buffered saline (PBS) buffer for 1 minute 3 times. Lysates were prepared using RIPA lysis buffer (Beyotime, Shanghai, China) with proteinase inhibitor (Roche, Basel, Switzerland), after shaking on ice for 30 minutes, the cells were harvested and centrifuged at 15,000 × g for 30 min. Rabbit anti-HA antibody (Santa Cruz) was mixed with the protein-G-agarose beads (30 μl) in the supernatant at 4°C overnight. The beads were prepared by centrifugation and washed three times with RIPA lysis buffer. Proteins binding to the beads were eluted by adding 30 μl of 2× SDS sample buffer and analyzed by immunoblotting using an anti-ubiquitin antibody (Santa Cruz).

### Chromatin immunoprecipitation PCR (ChIP-PCR)

For ChIP analysis, GFP, GFP-Gfi1aa or GFP-Gfi1aa Δzinc finger mutant expressing embryos were harvested at 48 hpf for brief fixation. Cross-linked chromatin was immunoprecipitated with anti-GFP antibody according to the procedure described [55]. The resultant immunoprecipitated samples were subjected to quantitative PCR using primer pairs (S1 Table).

### Cell line and treatment

HL60 cells were maintained in 1640 (Life technologies, Grand Island, NY, USA) with 10% Fetal Bovine Serum (Life technologies, Grand Island, NY, USA). HL60 cells were treated with 1 μM of ATRA (Sigma Aldrich) for 48 and 72 hours.

### Statistical analysis

Data were analyzed by SPSS software (version 20) using two tailed Student t test for comparisons between two groups and one-way analysis of variance (ANOVA) among multiple groups. Differences were considered significant at P<0.05. Data are expressed as mean ± standard error of the mean (SEM).

## Supporting information

S1 Fig Expression of lineage specific markers during primitive and definitive hematopoiesis stages in *irf2bp2a*-deficient embryos. (A-C’) WISH analyses of neutrophil markers *c/ebp1* (A, A’), *mpx* (B, B’), and early embryonic macrophage marker *mfap4* (C, C’) at 22 hpf in RBI in wild type (WT) and *irf2bp2a*-deficient embryos, respectively. n/n, number of embryos showing representative phenotype/total number of embryos examined. (D-H’) WISH analyses of *scl* (the key transcription factor initiating primitive hematopoiesis) (D, D’), erythroid markers *gata1* (E, E’), *hbαe1* (F, F’), *band3* (G, G’) and *alas2* (H-H’) in ICM at 22 hpf. (I-M’) WISH analyses of erythroid marker *hbαe1* (I, I’), monocyte and macrophage markers *mfap4* (J, J’) and *csf1r* (K, K’), lymphoid marker *rag1* (L, L’), HSPC marker *c-myb* (M, M’) in VDA and CHT from 2 dpf to 5 dpf. (N) Statistical results for A-C’, J-K’, and M-M’ (Student t test, N = 5, 15–27 embryos were used for each experiment. Error bars represent mean ± SEM. ns: not statistically significant).
(TIF)

S2 Fig Quantitative reverse transcriptase polymerase chain reaction analysis of neutrophil differentiation-related genes including *c/ebpα*, *c/ebp1*, *pu*.*1*, *mpx*, and *lyz* in GFP positive cells enriched from Tg(*mpx*:eGFP) and *irf2bp2a*^*-/-*^//Tg(*mpx*:eGFP) WKM samples. To determine the relative expression rate, data were normalized to the expression level of WT groups (which were set to 1.0) after normalized to the internal control of *β-actin*. Student t test, N = 3. Error bars represent mean ± SEM. **P < 0.01, ***P < 0.001.
(TIF)

S3 Fig Expression of neutrophil markers in *irf2bp2a* MO injected embryos. (A-C’) WISH analyses of *mpx* (A-B’) and *lyz* (C, C’) in wild type (WT) and *irf2bp2a* MO injected embryos. n/n, number of embryos showing representative phenotype/total number of embryos examined. (D) Statistical results for A-C’ (Student t test, N = 5, 19–25 embryos were used for each experiment. Error bars represent mean ± SEM. ***P < 0.001, ****P < 0.0001. (E, E’) WISH analyses of *mpx* in *irf2bp2a* MO injected embryos, *irf2bp2a* MO and *irf2bp2a* mRNA co-injection embryos. (F) Statistical results for E and E’ (Student t test, N = 5, 19–21 embryos were used for each experiment. Error bars represent mean ± SEM. *P < 0.1.
(TIF)

S4 Fig Quantitative reverse transcriptase polymerase chain reaction analysis of neutrophil differentiation-related genes including *c/ebpα*, *c/ebp1*, *pu*.*1*, *mpx*, and *lyz* in GFP positive cells enriched from Tg(*mpx*:eGFP) and *gfi1aa* MO injected Tg(*mpx*:eGFP) embryos at 2 dpf. To determine the relative expression rate, data were normalized to the expression level of WT groups (which were set to 1.0) after normalized to the internal control of *β-actin*. Student t test, N = 3. Error bars represent mean ± SEM. **P < 0.01, ***P < 0.001, ****P < 0.0001.
(TIF)

S5 Fig Representative scatterplot generated by FACS analysis of WKM samples collected from WT and *irf2bp2a*^*-/-*^ lines in 4-month-old adults. Student t test, N = 5. Error bars represent mean ± SEM. ns: not statistically significant.
(TIF)

S6 Fig Western blot analysis of FLAG-IRF2BP2a, FLAG-IRF2BP2b and HA-GFI1 expressing HEK293 cells. The proteasome inhibitor MG132 (2.5 μM) was used to inhibit the degradation of ubiquitinated proteins. Equal protein amounts for each sample were loaded (anti-ACTIN).
(TIF)

S7 Fig Expression of *irf2bp2a* and *irf2bp2b* transcript in HL60 cells treated with ATRA. *β-actin* served as the internal control. The expression levels of 48 h and 72 h groups were normalized to that of 0 h. Student t test, N = 3. Error bars represent mean ± SEM. *P < 0.1, **P < 0.01, ***P < 0.001.
(TIF)

S8 Fig Western blot analysis of FLAG-IRF2BP2a, HA-GFI1 and HIS-TRIAD1 expressing HEK293 cells. The proteasome inhibitor MG132 (2.5 μM) was used to inhibit the degradation of ubiquitinated proteins. Equal protein amounts for each sample were loaded (anti-ACTIN).
(DOCX)

S1 Table Primers for plasmid generation, luciferase assays, quantitative PCR, and ChIP-qPCR. (XLSX)
