# Supplementary figures and images for: Irf2bp2a regulates terminal granulopoiesis through proteasomal degradation of Gfi1aa in zebrafish
Source: PLoS Genet. 2021 Aug 5;17(8):e1009693. doi: 10.1371/journal.pgen.1009693 (PMC8370619; doi:10.1371/journal.pgen.1009693)

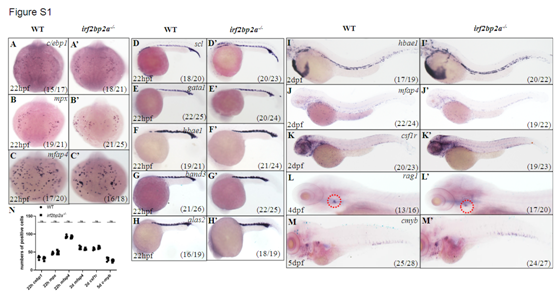

Supplement: S1 Fig — (A-C’) WISH analyses of neutrophil markers c/ebp1 (A, A’), mpx (B, B’), and early embryonic macrophage marker mfap4 (C, C’) at 22 hpf in RBI in wild type (WT) and irf2bp2a-deficient embryos, respectively. n/n, number of embryos showing representative phenotype/total number of embryos examined. (D-H’) WISH analyses of scl (the key transcription factor initiating primitive hematopoiesis) (D, D’), erythroid markers gata1 (E, E’), hbαe1 (F, F’), band3 (G, G’) and alas2 (H-H’) in ICM at 22 hpf. (I-M’) WISH analyses of erythroid marker hbαe1 (I, I’), monocyte and macrophage markers mfap4 (J, J’) and csf1r (K, K’), lymphoid marker rag1 (L, L’), HSPC marker c-myb (M, M’) in VDA and CHT from 2 dpf to 5 dpf. (N) Statistical results for A-C’, J-K’, and M-M’ (Student t test, N = 5, 15–27 embryos were used for each experiment. Error bars represent mean ± SEM. ns: not statistically significant). (TIF) [file pgen.1009693.s001.tif]

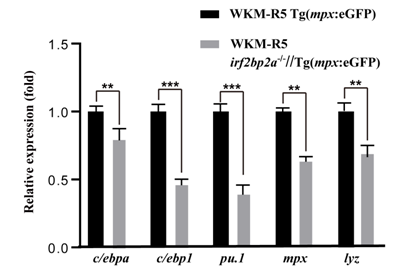

Supplement: S2 Fig — To determine the relative expression rate, data were normalized to the expression level of WT groups (which were set to 1.0) after normalized to the internal control of β-actin. Student t test, N = 3. Error bars represent mean ± SEM. **P < 0.01, ***P < 0.001. (TIF) [file pgen.1009693.s002.tif]

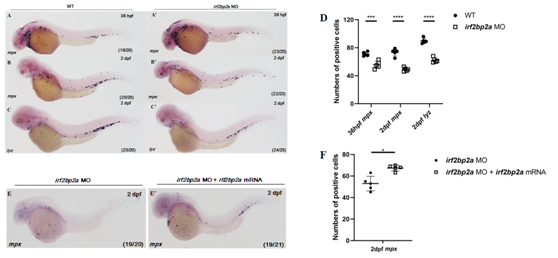

Supplement: S3 Fig — (A-C’) WISH analyses of mpx (A-B’) and lyz (C, C’) in wild type (WT) and irf2bp2a MO injected embryos. n/n, number of embryos showing representative phenotype/total number of embryos examined. (D) Statistical results for A-C’ (Student t test, N = 5, 19–25 embryos were used for each experiment. Error bars represent mean ± SEM. ***P < 0.001, ****P < 0.0001. (E, E’) WISH analyses of mpx in irf2bp2a MO injected embryos, irf2bp2a MO and irf2bp2a mRNA co-injection embryos. (F) Statistical results for E and E’ (Student t test, N = 5, 19–21 embryos were used for each experiment. Error bars represent mean ± SEM. *P < 0.1. (TIF) [file pgen.1009693.s003.tif]

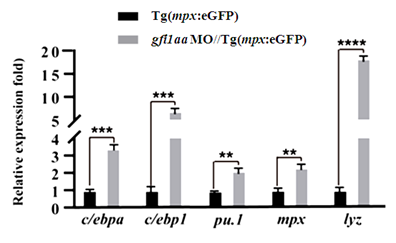

Supplement: S4 Fig — To determine the relative expression rate, data were normalized to the expression level of WT groups (which were set to 1.0) after normalized to the internal control of β-actin. Student t test, N = 3. Error bars represent mean ± SEM. **P < 0.01, ***P < 0.001, ****P < 0.0001. (TIF) [file pgen.1009693.s004.tif]

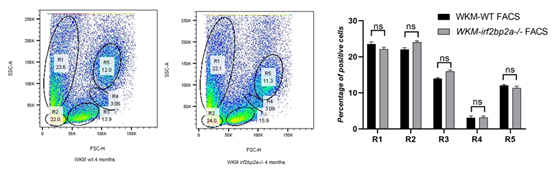

Supplement: S5 Fig — Student t test, N = 5. Error bars represent mean ± SEM. ns: not statistically significant. (TIF) [file pgen.1009693.s005.tif]

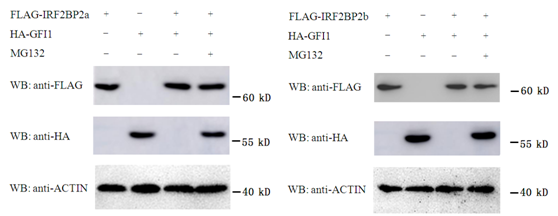

Supplement: S6 Fig — The proteasome inhibitor MG132 (2.5 μM) was used to inhibit the degradation of ubiquitinated proteins. Equal protein amounts for each sample were loaded (anti-ACTIN). (TIF) [file pgen.1009693.s006.tif]

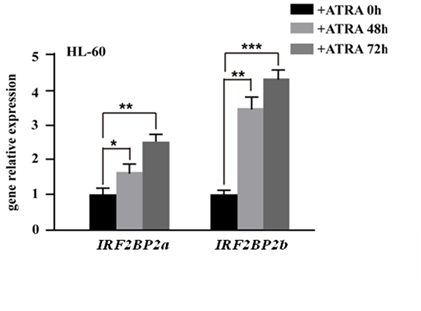

Supplement: S7 Fig — β-actin served as the internal control. The expression levels of 48 h and 72 h groups were normalized to that of 0 h. Student t test, N = 3. Error bars represent mean ± SEM. *P < 0.1, **P < 0.01, ***P < 0.001. (TIF) [file pgen.1009693.s007.tif]
