# Supplementary material for: Irf2bp2a regulates terminal granulopoiesis through proteasomal degradation of Gfi1aa in zebrafish
Source: PLoS Genet. 2021 Aug 5;17(8):e1009693. doi: 10.1371/journal.pgen.1009693 (PMC8370619; doi:10.1371/journal.pgen.1009693)
Supplement: S8 Fig — The proteasome inhibitor MG132 (2.5 μM) was used to inhibit the degradation of ubiquitinated proteins. Equal protein amounts for each sample were loaded (anti-ACTIN). (DOCX) [file pgen.1009693.s008.docx]

**S8_Fig. Western blot analysis of FLAG-IRF2BP2a, HA-GFI1 and HIS-TRIAD1 expressing HEK293 cells.** The proteasome inhibitor MG132 (2.5 μM) was used to inhibit the degradation of ubiquitinated proteins. Equal protein amounts for each sample were loaded (anti-ACTIN).
